# Development of Cerebral Microbleeds in the APP23-Transgenic Mouse Model of Cerebral Amyloid Angiopathy—A 9.4 Tesla MRI Study

**DOI:** 10.3389/fnagi.2016.00170

**Published:** 2016-07-08

**Authors:** Björn Reuter, Alexander Venus, Patrick Heiler, Lothar Schad, Anne Ebert, Michael G. Hennerici, Saskia Grudzenski, Marc Fatar

**Affiliations:** ^1^Department of Neurology and Neurophysiology, Freiburg UniversityFreiburg, Germany; ^2^Department of Neurology, Universitätsmedizin Mannheim, Heidelberg UniversityMannheim, Germany; ^3^Computer Assisted Clinical Medicine, Heidelberg UniversityMannheim, Germany

**Keywords:** amyloid, CAA, cerebral microbleeds, transgenic mice, APP23, MRI

## Abstract

**Background**: Cerebral amyloid angiopathy (CAA) is characterized by extracellular deposition of amyloid β (Aβ) around cerebral arteries and capillaries and leads to an increased risk for vascular dementia, spontaneous lobar hemorrhage, convexal subarachnoid hemorrhage, and transient focal neurological episodes, which might be an indicator of imminent spontaneous intracerebral hemorrhage. In CAA cerebral microbleeds (cMBs) with a cortical/juxtacortical distribution are frequently observed in standard magnetic resonance imaging (MRI). *In vivo* MRI of transgenic mouse models of CAA may serve as a useful tool to investigate translational aspects of the disease.

**Materials and Methods**: APP23-transgenic mice demonstrate cerebrovascular Aβ deposition with subsequent neuropathological changes characteristic for CAA. We performed a 9.4 Tesla high field MRI study using T2, T2* and time of flight-magnetic resonance angiograpy (TOF-MRA) sequences in APP23-transgenic mice and wildtype (wt) littermates at the age of 8, 12, 16, 20 and 24 months, respectively. Numbers, size, and location of cMBs are reported.

**Results**: T2* imaging demonstrated cMBs (diameter 50–300 μm) located in the neocortex and, to a lesser degree, in the thalamus. cMBs were detected at the earliest at 16 months of age. Numbers increased exponentially with age, with 2.5 ± 2 (median ± interquartilrange) at 16 months, 15 ± 6 at 20 months, and 31.5 ± 17 at 24 months of age, respectively.

**Conclusion**: We report the temporal and spatial development of cMBs in the aging APP23-transgenic mouse model which develops characteristic pathological patterns known from human CAA. We expect this mouse model to serve as a useful tool to non-invasively monitor mid- and longterm translational aspects of CAA and to investigate experimental therapeutic strategies in longitudinal studies.

## Introduction

Cerebral amyloid angiopathy (CAA) causes a higher risk for spontaneous lobar hemorrhage and cognitive decline (Yamada, 2015). Although the incidence of CAA increases constantly with age, there is no clear association with the common cerebrovascular risk factors. The prevalence in the elderly population ranges from 28 to 38% in non-demented to 55–59% in demented subjects (Keage et al., 2009). Amyloid beta (Aβ) as the main pathological substrate travels along pericapillary interstitial pathways before it aggregates and deposits more in the arterial than in the capillary system (Smith and Greenberg, 2009). Experimental and histopathological studies suggest that vascular accumulation of Aβ is not only a result of enhanced production of this peptide, but also of its reduced clearance from the brain interstitial fluid into the blood across the blood brain barrier (BBB; Weller et al., 2008). Histological findings in CAA are well known for several decades and comprise of degenerated vascular smooth muscle cells, loss of tight junction proteins, clustered occurrence of activated microglial cells, infiltration of leucocytes, fibrinoid necrosis, double barreling and microaneurysms (Scholz, 1938; Smith and Greenberg, 2009; Carrano et al., 2012).

Magnetic resonance imaging (MRI) as the brain imaging technique of choice demonstrates distinctive features as cerebral microbleeds (cMBs) predominantly located within the cortex and subcortex, white matter changes, superficial siderosis/convexal subarachnoid hemorrhage, silent cortical infarcts, inflammatory disease, and atypical lobar hemorrhage (Chao et al., 2006). Among these neuroimaging findings, cMBs have received the highest attention, being frequently observed in T2* gradient echo sequences and susceptibility-weighted imaging (SWI; Haacke et al., 2007). They represent focal deposits of perivascular hemosiderin-iron and their distribution pattern in synopsis of further imaging findings may allow for etiologic conclusions, i.e., CAA, severe arterial hypertension, chronic cerebral infarction, head trauma, genetic vessel disease, or endocarditis (Schrag and Greer, 2014). In CAA they are closely connected to Aβ deposits in arterioles and, to a lesser extent, capillaries (Schrag et al., 2010; Park et al., 2013). Focal rupture of the vessel wall is thought to be a consequence of Aβ-induced inflammation and loss of vascular smooth muscle cells (Schrag et al., 2010). Strictly lobar cMBs were positively associated with amyloid burden in Pittsburgh compound B (PiB) positron emission tomography (PET), cognitive function and the risk for spontaneous lobar hemorrhage (Poels et al., 2012; Park et al., 2013; van Etten et al., 2014).

The development of transgenic mice was essential to understand the pathogenesis of Alzheimer’s disease (AD) and CAA and to investigate promising therapeutic strategies. While there are several different transgenic mouse lines which represent the pathological correlate of AD, only few can serve as models for CAA. Those are in particular the APP23, Tg2576, APPDutch, ArcAβ and TgSwDi mouse lines (Hsiao et al., 1996; Calhoun et al., 1999; Davis et al., 2004; Herzig et al., 2004; Elder et al., 2010; Klohs et al., 2011). Histological validation of a therapeutical approach in transgenic mouse models is essential to understand the underlying mechanisms. However, we believe that under translational aspects a non-invasive brain imaging with methods well-established in clinical routine might be of high value. Therefore, we conducted a high-field 9.4 Tesla MRI-study in APP23-tg mice and wildtype (wt) littermates with the objective to characterize the temporal and spatial development of cMBs. Based on the presented data we regard cMBs in the APP23-tg mouse model useful as an outcome marker for preclinical testing of pharmatherapeutical approaches under standardized conditions, which under methodological aspects can also be easily transferred and replicated in human clinical trials.

## Materials and Methods

### Animals

APP23-tg mice contain a human amyloid precursor protein (APP751) cDNA with the Swedish double mutation at position 670/671 under the control of the neuron-specific Thy-1 promoter (Calhoun et al., 1999). These animals express APP in sevenfold excess compared with the endogenous murine APP. Histologically visible parenchymal Aβ deposition starts at 6–8 months of age. Mainly affected are the neocortex, hippocampus, and thalamus (Sturchler-Pierrat et al., 1997). Besides to parenchymal deposition the mouse model shows a substantial cerebrovascular accumulation of Aβ, first detectable in histology at the age of 12 months (Calhoun et al., 1999; Sturchler-Pierrat and Staufenbiel, 2000). Leakage of the BBB, cerebral microhemorrhages, and macrohemorrhages are closely connected to the affected vessels and show similarities to human pathologies (Winkler et al., 2001; Beckmann et al., 2003).

Approval of our experiments was given by the local ethical animal care and use committee (Regierungspräsidium Karlsruhe, 76247 Karlsruhe, Germany, file number 35-9185.81/G-9/10). All procedures were in strict accordance with institutional animal protection guidelines. Heterozygote B6, D2-TgN[Thy-APPSWE]-23-tg mice (APP23-tg) provided by Matthias Staufenbiel (Novartis Institutes for BioMedical Research, Novartis Pharma AG, Basel, Switzerland) were backcrossed twice with C57BL/6 mice (Janvier, Saint Berthevin Cedex, France) and kept under a 12/12 h light/dark cycle with standard food and water *ad libitum*. Fourty-eight heterozygote mice and 48 wt littermates were bred to reach a final study cohort of 60 mice and compensate for spontaneous death. APP23-tg mice and wt controls (*n* = 6 each) were measured at the age 8, 12, 16, 20 and 24 months, respectively. No further in- or exclusion criteria were applied and mice of both sexes were used in our study. Wt mice do not develop spontaneous cMBs (Klohs et al., 2011; Hoffmann et al., 2016). With inclusion of wt littermates we aimed to identify possible MRI abnormalities morphologically similar to cMBs, which have to be taken into account for cMB counting in APP23-tg mice.

### Magnetic Resonance Imaging (MRI) Protocol and Analysis

*In vivo* brain imaging was performed in 4-month intervals within an age range of 8–24 months and consisting of six mice each. For imaging a 9.4 T Biospec 94/20 USR small animal system equipped with 740 mT/m gradients and a 1H surface cryogenic probe (Bruker, Ettlingen, Germany) was used as described before (Reuter et al., 2014). T2*-weighted gradient echo images were used to demonstrate hemosiderin deposits resulting from cMBs. SWI with its higher sensitivity to detect cMBs in humans has been previously described to be impractical for rodent *in vivo* imaging due to susceptibility interface-related signal loss in the cortex (Chamberlain et al., 2009). We’ve tested for SWI and faced the same problem of artifacts in the air/brain tissue border zones, which interfered with sufficient evaluation of cortical cMBs.

Twelve coronary sections covering the whole brain were analyzed. Hypointense regions in T2*-weighted images considered to be cMBs were verified by comparison to time of flight-magnetic resonance angiograpy (TOF-MRA) raw data to distinguish vessel related flow void. cMBs were quantified and graded *in vivo* in APP23-tg and wt littermates depending on size (cMBs with size ≤100 μm, 150–200 μm or >200 μm) and spatial distribution (cortex and thalamus). Age-matched wt mice served as controls to assess the frequency of artifacts susceptive for cMBs. The quantification and size-grading of cMBs was performed by an investigator blinded for age and genetic status.

### Histology

Histology was done as described previously (Reuter et al., 2016). In short, animals were sacrificed within 3 days after MRI under deep Isoflurane anesthesia by transcardial perfusion with 4% acid free formalin (Roth, Karlsruhe, Germany). The harvested brains were incubated over night in 4% acid free formalin at 4°C, cut into blocs with 2 mm thickness, dehydrated with ethanol and xylol and embedded in paraffin. For histochemical analysis 4 μm sections were dewaxed in xylene and rehydrated in alcohol and distilled water.

For detection of cMBs Prussian blue (PB) staining was performed using the Accustain® Iron Stain Kit as described in the manufacturer’s protocol (Sigma-Aldrich, St. Louis, MO, USA). Nuclei were counterstained using nuclear fast red 0.1% (Merck, Darmstadt, Germany) for 10 min. Following dehydration steps in alcohol and xylol the sections were preserved in mounting medium (Eukitt, O. Kindler, Freiburg, Germany).

Bright field analysis was done using a Leica DM 4500 B fluorescence microscope (Leica, Wetzlar, Germany). Pictures were taken with Leica IM50 Image Manager Software (Leica, Cambridge, UK).

### Statistical Analysis

Statistical analysis was performed with a standard software package (SPSS 22, “SPPS Inc.”, Chicago, IL, USA). The statistical evaluation was performed using univariate analysis of variance. After significant analyses of variance, multiple *post hoc* comparisons were carried out using the Scheffé test. Data were visualized with boxplots and expressed as median and interquartile range. A *p* < 0.05 was considered significant.

## Results

### Spatial Distribution of cMBs Depending on Age

**Figure 1:**
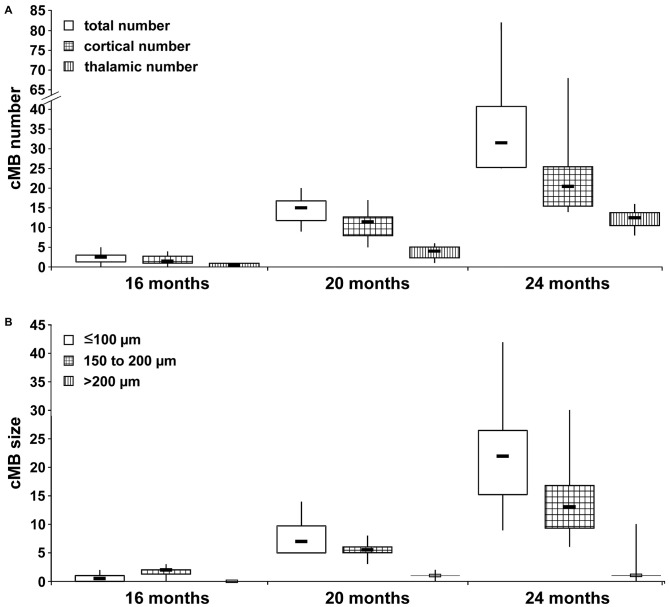
**Spatial and size distribution of cerebral microbleeds (cMBs) depending on age. (A)** Total cMB numbers and the amount of cMBs located in cortex and thalamus were obtained for APP23-tg mice aged 8, 12, 16, 20 and 24 months. Since APP-tg mice aged 8 and 12 months did not display cMBs only values for APP-tg mice aged 16, 20 and 24 months (each group *n* = 6) are shown. **(B)** cMBs were graded depending on size (≤100 μm, 100–150 μm and >200 μm) for APP-tg mice aged 16, 20 and 24 months (each group *n* = 6).

**Figure 2:**
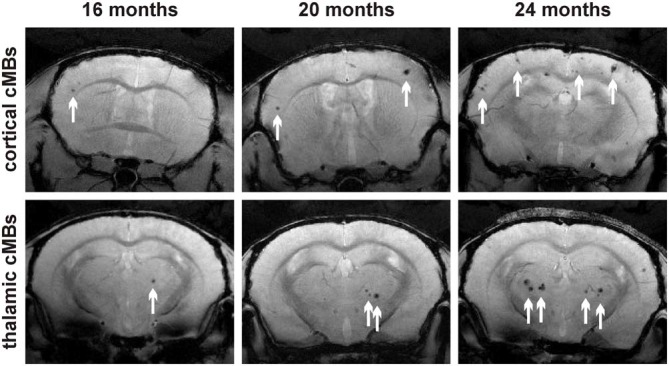
Representative T2* magnetic resonance images of cortical and thalamic cMBs in the APP23-tg mouse model at 16, 20, and 24 months of age.

**Figure 3:**
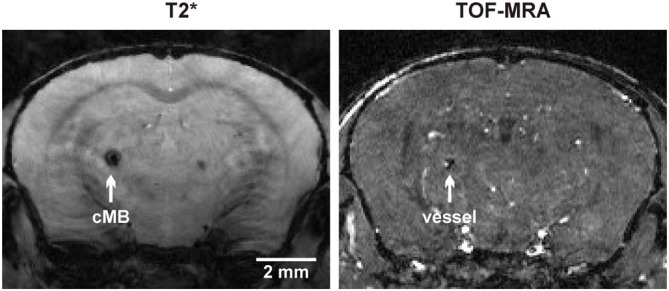
Colocalization of a thalamic cMB and an adjacent arterial vessel using T2* imaging and time of flight-magnetic resonance angiograpy (TOF-MRA).

**Figure 4:**
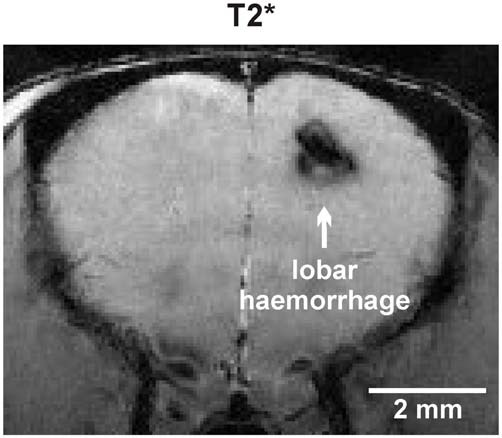
**Spontaneous lobar intracerebral hemorrhage in the left hemispheric frontal lobe of an APP23-tg mouse 20 months of age**.

Total cMB numbers as well as the amount of cMBs located in cortex and thalamus were obtained for each age-group (Figure 1A, Supplementary Table S1). Results showed that up to 12 months APP23-tg mice displayed no cMBs. Starting at 16 months total cMB numbers increased exponentially with age (*p* < 0.001). Approximately two thirds of the cMBs were located in the cortex and one third in the thalamus. Representative images on cMBs in cortical and thalamic location at the age of 16, 20, and 24 months are shown in Figure 2. Rarely an adjacent arterial vessel was observed in TOF-MRA images and one mouse 20 months of age displayed a spontaneous lobar hemorrhage in the left hemispheric frontal cortex (Figures 3, 4, respectively). Wt littermates displayed a low background level of hypointense focal lesions of unclassified origin first observed at the age of 16 months, where the presence of cMBs could not be fully excluded by neuroimaging. Due to their insignificant frequency no adjustment of cMB counts in APP23-tg mice is regarded necessary.

### Size Distribution of cMBs Depending on Age

Numbers of cMBs with size of ≤100 μm, 150–200 μm or >200 μm were obtained for each age-group (Figure 1B, Supplementary Table S2). At the age of 16 months cMBs were at maximum 200 μm in diameter. With increasing age small cMBs (cMBs ≤100 μm) were observed in highest frequency (median 22, IQR 13 at the age of 24 months, *p* < 0.001) followed by cMBs 150–200 μm in diameter (median 13, IQR 8 at 24 months, *p* < 0.001), whereas the frequency of large cMBs >200 μm was generally low and no association with age was observed (median 1, IQR 0 at 24 months, *p* = 0.1). Hypointense focal lesions in wt mice were generally small ≤100 μm in diameter and their frequency was independent from age (*p* = 0.2).

### Histological Matching of MRI Findings

**Figure 5:**
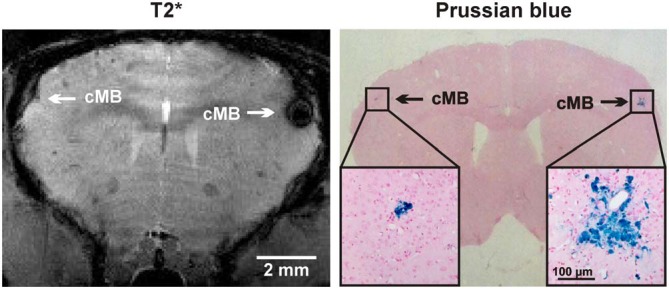
Comparison of cMBs in the APP23-tg mouse model using T2* magnetic resonance imaging (MRI) and Prussian blue (PB) staining. In MRI the detection of the right hemispheric cMB was hindered by an artifact but approved in the neighboring T2* sequence and TOF-MRA raw data.

Following brain imaging a neuropathologic examination was performed in a 24 month old APP23-tg mouse for spatial colocalization of cMBs (Figure 5). Using T2* imaging sequences as reference a matched histological section with PB staining is demonstrated.

## Discussion

Besides slowly progressing CAA-related syndromes, i.e., vascular dementia and gait disturbances, cerebrovascular events as spontaneous lobar intracerebral hemorrhage (ICH), convexal subarachnoid hemorrhage and the very recently discovered CAA-related inflammation require immediate hospitalization. Acute therapy nowadays comprises of admission to organized stroke units or intensive care units and treatment strategies not specifically related to CAA. This includes the consideration of hematoma evacuation in lobar ICH, immunosuppressive therapy in CAA-related inflammation and avoidance of immobility-related complications with early mobilization and rehabilitation strategies (Steiner et al., 2014; AVERT Trial Collaboration Group, 2015; Raghavan et al., 2016). Since the incidence of CAA and subsequent vascular disease is expected to increase in aging societies, strong efforts are undertaken to develop new treatment options. These strategies are closely related to therapy approaches in AD, which have received a much higher public and financial support for many decades. However, in CAA they try to target the vascular accumulation of Aβ itself and/or aim to stabilize vascular function (Saito and Ihara, 2014; Reuter et al., 2016). A second aspect of growing interest are safety concerns regarding CAA patients in need for either acute ischemic stroke treatment with intravenous recombinant tissue plasminogen activator (rtPA) or long-term treatment with platelet aggregation inhibitors and/or oral anticoagulants (Reuter et al., 2014; Charidimou et al., 2015a; Wilson et al., 2015). cMBs are one of the most important clinicoradiological findings for risk stratification prior to treatment with either rtPA or antithrombotics.

We present a transgenic mouse model of CAA which develops cMBs at the earliest between 12–16 months of age. The majority of cMBs detected in T2*-weighted imaging are 50–200 μm in size, while cMBs larger than 200 μm were less frequently observed. About two thirds of the cMBs are located in the neocortex and, contrary to human disease, about one third in the thalami. APP23-tg mice are known to develop severe CAA in thalamic vessels (Thal et al., 2009). Since they do not connect to human CAA it thus might be suitable in experimental studies to focus solely on cortical cMBs. The overall burden of cMBs showed a significant and exponential increase over time with median numbers ranging from 2.5 in 16 months old mice to 31.5 in 24 months old mice. However, in aged mice an increasing variability in numbers was observed, with an IQR of 2 at 16 months and 17 at 24 months of age. Previous work has reported an age-dependent exponential increase of cMBs in this mouse model using histology or T2*-weighted MRI at 4.7 T field intensity, respectively. In histology the mean number of cMBs at 27 months of age was reported to be 14 (right hemisphere only, every 10th section, total estimated cMB load >100), and a positive correlation with CAA-severity was observed (Winkler et al., 2001). In MRI at 4.7 T the mean number of discovered cMBs at the age of 24 months was reported 5 compared to 31.5 in our study at 9.7 T field intensity, thereby demonstrating the technical improvement of current non-contrast enhanced rodent MRI (Beckmann et al., 2011). Compared to the gold standard histology T2* high field MRI now offers a valid option to rapidly and serially determine the whole brain cMB load *in vivo*. Using the same MRI protocol, we’ve recently demonstrated a good correlation between MRI and histology-derived numbers of cMBs in the APP23-tg mouse model and thus the validity of MRI derived data (Reuter et al., 2016).

Prior to clinical trials novel agents need to prove their efficacy and safety in animal models of CAA and transgenic mouse models are regarded invaluable for this undertaking. Moreover, in a retrograde approach transgenic mouse models may pathophysiologically explain findings made from observational or clinical studies. There are several possibilities to invasively and non-invasively monitor the progression of CAA, i.e., histological analysis with Congo Red, Thioflavin S or immunostaining, brain imaging with MRI, PiB PET, and light-based microscopy methods, i.e., two-photon microscopy or optical coherence tomography (for detailed information, see Klohs et al., 2014). Compared to other imaging parameters, cMBs in cortical distribution are frequently observed in patients suffering CAA. Although cMBs are not a clinical outcome marker *per se*, their relationship to morphological and clinical CAA severity and progression and their high incidence in standard non-invasive brain imaging make them sufficiently sensitive and specific as an outcome parameter for clinical trials (Greenberg et al., 2014; Charidimou et al., 2015b). Therefore, the longitudinal assessment of cMB numbers is regarded to be a useful tool to non-invasively monitor CAA progression, which in the future could be further improved by 3D techniques at very high spatial resolution (Greenberg et al., 2014; Chacon-Caldera et al., 2016). Moreover, in many healthcare systems cranial MRI scanners including gradient echo sequences or susceptibility weighted imaging are widely available and offer a good opportunity for safety monitoring in phase IV clinical trials, when a therapeutic agent has received approval for CAA. With our study we demonstrate that cMBs in the APP23-tg mouse model are easily and reliably detectable using whole brain high field 9.4 Tesla MRI. cMBs are not uniquely observed in APP23-tg mice but have also been described in Tg2576, TgSwDI and arcAβ mice (Fisher et al., 2011; Klohs et al., 2011; Yang et al., 2011). Yet to the best of our knowledge, we provide the most comprehensive description of cMBs regarding their incidence in ageing, their distribution, and their size. Based on the numbers of cMBs observed at 24 months of age the following sample sizes were calculated to detect a significant reduction of cMBs in experimental trials with two groups. Power analysis was performed for a single-tailed two-group independent sample *t*-test (α 0.05, β 0.80). Regarding a therapeutical approach with an assumed large effect size (*d* = 0.80) *N* = 21 mice per group are required and for a medium effect size (*d* = 0.50) *N* = 51 per group. In case research groups aim to investigate other age cohorts or cMBs in cortical or thalamic localization only the raw data is provided as a supplementary file (Supplementary Table S3) to perform the respective power analyses. We hope that this data facilitates the conduction of future experimental studies with the APP23-tg mouse model.

Current therapeutical approaches are closely connected to AD research. Possible targets to reduce the vascular burden of Aβ are proteolytic pathways, uptake and degradation by microglial cells and astrocytes, the modulation of Aβ efflux and influx over the BBB, and finally the stimulation of perivascular drainage alongside of small arteries and arterioles within the brain interstitial fluid (Charidimou et al., 2012; Saito and Ihara, 2014). In future studies we intent to investigate the effect of ligand stimulated nuclear receptors (NR) on Aβ secretion and APP processing in APP23-tg mice. NRs are ligand-activated transcription factors providing a critical role between the genome and the environment (Mandrekar-Colucci and Landreth, 2011). It was shown that drugs targeting NRs may strongly influence the regulation of cholesterol efflux and generation of high density lipoproteins (HDL), thus leading to diminished Aβ secretion (Riddell et al., 2007; Fitz et al., 2010; Sandoval-Hernández et al., 2015). Furthermore it has been shown that different mouse strains of AD including APP23-tg mice being deficient for NR targeted genes present altered Aβ levels (Hirsch-Reinshagen et al., 2005; Koldamova et al., 2005).

## Conclusion

In a longitudinal MRI study we demonstrate that APP23 transgenic mice develop cMBs at the earliest between 12 and 16 months of age. As in human CAA cMBs are located within the neocortex but contrary to human disease to a lesser degree also in the thalami. The overall burden of cMBs exponentially increases with age but also shows an increasing inter-individual variability in numbers. This needs to be taken into account for sample size calculation in experimental studies. Under translational aspects we expect this mouse model and method to serve as a useful tool to non-invasively monitor the development of CAA and to investigate experimental therapeutic strategies in longitudinal studies. Possible pitfalls comprise of the great variability of cMB prevalence in aged mice, which require careful consideration of the optimal timing of brain imaging and also a sufficient size of the study cohort.

## Author Contributions

BR, MGH, SG, and MF conceived and designed the experiments; BR, AV, and SG performed the experiments; BR, SG, and MF analyzed the data; PH and LS contributed analysis tools; BR wrote and submitted the article. All authors agree to be accountable for all aspects of the work and gave final approval for this version to be published.

## Funding

The study was funded by an internal grant of the Medical Faculty of Mannheim, University of Heidelberg.

## Supplementary Material

The Supplementary Material for this article can be found online at: http://journal.frontiersin.org/article/10.3389/fnagi.2016.00170/abstract

## Conflict of Interest Statement

The authors declare that the research was conducted in the absence of any commercial or financial relationships that could be construed as a potential conflict of interest.
