# Supplementary material for: Development of Cerebral Microbleeds in the APP23-Transgenic Mouse Model of Cerebral Amyloid Angiopathy—A 9.4 Tesla MRI Study
Source: Front Aging Neurosci. 2016 Jul 8;8:170. doi: 10.3389/fnagi.2016.00170 (PMC4937037; doi:10.3389/fnagi.2016.00170)
Supplement: Supplementary file 1 [file Table_1.DOCX]

Supplementary Material

Development of cerebral microbleeds in the APP23-transgenic mouse model of cerebral amyloid angiopathy - a 9.4 Tesla MRI study

Björn Reuter^*^, Alexander Venus, Patrick Heiler, Lothar Schad, Anne Ebert, Michael G. Hennerici, Saskia Grudzenski^#^; Marc Fatar^#^

*** Correspondence:** Björn Reuter: bjoern.reuter@uniklinik-freiburg.de

| **Supplementary table S1:** Total numbers and spatial distribution of cerebral microbleeds. | | | | | | | | |
| --- | --- | --- | --- | --- | --- | --- | --- | --- |
| Variable | | APP23 transgenic mice (n=30) | | |  | wildtype controls (n=30) | | |
| Age (months) | | total cMBs, median (IQR) | cortical cMBs, median (IQR) | thalamic cMBs, median (IQR) |  | total cMBs, median (IQR) | cortical cMBs, median (IQR) | thalamic cMBs, median (IQR) |
|  | 8 | 0 | 0 | 0 |  | 0 | 0 | 0 |
|  | 12 | 0 | 0 | 0 |  | 0 (1) | 0 (1) | 0 |
|  | 16 | 2.5 (2) | 1.5 (2) | 0.5 (1) |  | 0.5 (1) | 0 (1) | 0 (1) |
|  | 20 | 15 (6) | 11.5 (6) | 4 (3) |  | 1 (1) | 1 (1) | 0.5 (1) |
|  | 24 | 31.5 (17) | 20.5 (11) | 12.5 (4) |  | 1 (2) | 0 (1) | 0 (1) |
| N=6 animals were assessed at each time point. The interquartile range was indicated were appropriate. Abbreviations: cMBs, cerebral microbleeds; IQR, interquartile range. | | | | | | | | |
