# Supplementary material for: Development of Cerebral Microbleeds in the APP23-Transgenic Mouse Model of Cerebral Amyloid Angiopathy—A 9.4 Tesla MRI Study
Source: Front Aging Neurosci. 2016 Jul 8;8:170. doi: 10.3389/fnagi.2016.00170 (PMC4937037; doi:10.3389/fnagi.2016.00170)
Supplement: Supplementary file 3 [file Table_3.DOCX]

Supplementary Material

Development of cerebral microbleeds in the APP23-transgenic mouse model of cerebral amyloid angiopathy - a 9.4 Tesla MRI study

Björn Reuter^*^, Alexander Venus, Patrick Heiler, Lothar Schad, Anne Ebert, Michael G. Hennerici, Saskia Grudzenski^#^; Marc Fatar^#^

*** Correspondence:** Björn Reuter: bjoern.reuter@uniklinik-freiburg.de

| **Supplementary Table S3:** cerebral microbleeds (cMBs) in APP23 transgenic mice detected in 9.4 T MRI (raw data) | | | | | | | |
| --- | --- | --- | --- | --- | --- | --- | --- |
| **mouse N⁰** | **age (months)** | **total cMBs** | **cMB size** | | | **cMB localisation** | |
|  |  |  | **<= 100µm** | **150-200µm** | **>200** | **cortical** | **thalamic** |
| 1 | 8 | 0 | 0 | 0 | 0 | 0 | 0 |
| 2 | 8 | 0 | 0 | 0 | 0 | 0 | 0 |
| 3 | 8 | 0 | 0 | 0 | 0 | 0 | 0 |
| 4 | 8 | 0 | 0 | 0 | 0 | 0 | 0 |
| 5 | 8 | 0 | 0 | 0 | 0 | 0 | 0 |
| 6 | 8 | 0 | 0 | 0 | 0 | 0 | 0 |
| 7 | 12 | 0 | 0 | 0 | 0 | 0 | 0 |
| 8 | 12 | 0 | 0 | 0 | 0 | 0 | 0 |
| 9 | 12 | 0 | 0 | 0 | 0 | 0 | 0 |
| 10 | 12 | 0 | 0 | 0 | 0 | 0 | 0 |
| 11 | 12 | 0 | 0 | 0 | 0 | 0 | 0 |
| 12 | 12 | 0 | 0 | 0 | 0 | 0 | 0 |
| 13 | 16 | 1 | 0 | 1 | 0 | 1 | 0 |
| 14 | 16 | 3 | 1 | 2 | 0 | 2 | 1 |
| 15 | 16 | 3 | 1 | 2 | 0 | 3 | 0 |
| 16 | 16 | 5 | 2 | 3 | 0 | 4 | 1 |
| 17 | 16 | 0 | 0 | 0 | 0 | 0 | 0 |
| 18 | 16 | 2 | 0 | 2 | 0 | 1 | 1 |
| 19 | 20 | 16 | 9 | 5 | 2 | 11 | 5 |
| 20 | 20 | 9 | 5 | 3 | 1 | 7 | 2 |
| 21 | 20 | 20 | 14 | 6 | 0 | 17 | 3 |
| 22 | 20 | 17 | 10 | 6 | 1 | 12 | 5 |
| 23 | 20 | 14 | 5 | 8 | 1 | 13 | 1 |
| 24 | 20 | 11 | 5 | 5 | 1 | 5 | 6 |
| 25 | 24 | 82 | 42 | 30 | 10 | 68 | 14 |
| 26 | 24 | 25 | 19 | 6 | 0 | 17 | 8 |
| 27 | 24 | 25 | 14 | 10 | 1 | 15 | 10 |
| 28 | 24 | 26 | 9 | 17 | 1 | 14 | 12 |
| 29 | 24 | 42 | 25 | 16 | 1 | 26 | 16 |
| 30 | 24 | 37 | 27 | 9 | 1 | 24 | 13 |
